# PICH Supports Embryonic Hematopoiesis by Suppressing a cGAS‐STING‐Mediated Interferon Response

**DOI:** 10.1002/advs.202103837

**Published:** 2022-01-17

**Authors:** Xinwei Geng, Chao Zhang, Miao Li, Jiaqi Wang, Fang Ji, Hanrong Feng, Meichun Xing, Fei Li, Lingling Zhang, Wen Li, Zhihua Chen, Ian D. Hickson, Huahao Shen, Songmin Ying

**Affiliations:** ^1^ Department of Pharmacology and Department of Respiratory and Critical Care Medicine of the Second Affiliated Hospital Zhejiang University School of Medicine Key Laboratory of Respiratory Disease of Zhejiang Province Hangzhou Zhejiang 310009 China; ^2^ Key Laboratory of Respiratory Disease of Zhejiang Province Department of Respiratory and Critical Care Medicine Second Affiliated Hospital of Zhejiang University School of Medicine Hangzhou Zhejiang 310009 China; ^3^ Department of Anatomy Zhejiang University School of Medicine Hangzhou Zhejiang 310058 China; ^4^ International Institutes of Medicine the Fourth Affiliated Hospital of Zhejiang University School of Medicine Yiwu Zhejiang 322000 China; ^5^ Center for Chromosome Stability and Center for Healthy Aging Department of Cellular and Molecular Medicine University of Copenhagen Blegdamsvej 3B Copenhagen N 2200 Denmark; ^6^ State Key Laboratory of Respiratory Diseases Guangzhou Guangdong 510120 China

**Keywords:** cGAS‐STING, genomic stability, hematopoietic stem cells, Plk1‐interacting checkpoint helicase, type I interferons

## Abstract

The Plk1‐interacting checkpoint helicase (PICH) protein localizes to ultrafine anaphase DNA bridges in mitosis along with a complex of DNA repair proteins. Previous studies show PICH deficiency‐induced embryonic lethality in mice. However, the function of PICH that is required to suppress embryonic lethality in PICH‐deficient mammals remains to be determined. Previous clinical studies suggest a link between PICH deficiency and the onset of acquired aplastic anemia. Here, using *Pich* knock‐out (KO) mouse models, the authors provide evidence for a mechanistic link between PICH deficiency and defective hematopoiesis. Fetal livers from *Pich*‐KO embryos exhibit a significantly elevated number of hematopoietic stem cells (HSCs); however, these HSCs display a higher level of apoptosis and a much‐reduced ability to reconstitute a functional hematopoietic system when transplanted into lethally irradiated recipients. Moreover, these HSCs show an elevated cytoplasmic dsDNA expression and an activation of the cGAS‐STING pathway, resulting in excessive production of type I interferons (IFN). Importantly, deletion of *Ifnar1* or *cGAS* reverses the defective hematopoiesis. The authors conclude that loss of PICH results in defective hematopoiesis via cGAS‐STING‐mediated type I IFN production.

## Graphical abstract

PICH is a genome stability maintenance protein. It is shown that hematopoietic stem cells (HSCs) from *Pich* knock out embryos display an elevated apoptosis level, a reduced reconstitute capacity and an elevated cytoplasmic dsDNA and type I interferons (IFN) expression. Deletion of cGAS reverses the defective hematopoiesis. The authors conclude that PICH deficiency impaired HSCs via cGAS‐mediated IFNs production.

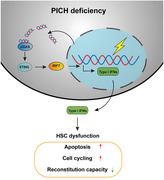

## 1 Introduction

Hematopoiesis is the process whereby all blood cell types are generated from hematopoietic stem cells (HSCs).^[^ ^1^ ^]^ In adult mice, this process occurs in the bone marrow; however, embryonic hematopoiesis occurs at different locations in mice, depending on the stage of embryonic development.^[^ ^2^ ^]^ Prior to embryonic day 10.5 (E10.5), primitive hematopoiesis occurs in the aorta‐gonad‐mesonephros (AGM) region.^[^ ^3^ ^]^ Following this, during the process of definitive hematopoiesis, the HSCs formed in the AGM region migrate to the fetal liver (FL) and undergo an expansion in number.^[^ ^4^, ^5^ ^]^ At E15.5, the HSCs enter the peripheral circulation, whereupon they home to the bone marrow.^[^ ^6^ ^]^ Embryonic hematopoiesis failure leads to severe developmental defects and embryonic lethality in severe cases.^[^ ^7^, ^8^ ^]^

Faithful maintenance of genome integrity in HSCs is crucial for successful hematopoiesis. Replication stress induced in stem cells by repeated rapid cell cycling is thought to be a major source of genome instability in HSCs. DNA repair deficiency results in progressive bone marrow failure (BMF) and cancer susceptibility.^[^ ^9^ ^]^ Most of inherited aplastic anemia (AA) cases are associated with DNA repair gene mutations, such as, dyskeratosis congenita (caused by mutations in *RTEL1*, *DKC1*, or *hTERC*)^[^ ^10^, ^11^, ^12^ ^]^ and Fanconi anemia (FA) (caused by mutations in any of the 22 FA genes, but most commonly *FANCA*, *FANCC* and *FANCG)*.^[^ ^13^, ^14^, ^15^, ^16^ ^]^ AA is a life‐threatening blood disorder, affecting children and adults. The pathophysiology of AA is via immune‐mediated destruction of bone marrow. Daria Babushok et al. conducted whole exome sequencing in 22 sporadic AA cases.^[^ ^17^ ^]^ Two patients exhibited missense mutations in *Pich*, a gene involved in chromosomal stability maintenance, suggesting a link between Plk1‐interacting checkpoint helicase (PICH) deficiency and defective hematopoiesis.

PICH is an SNF2 family DNA translocase that is a substrate for the key mitotic regulator, polo‐like kinase 1 (PLK1).^[^ ^18^, ^19^ ^]^ It has been reported that, during mitosis PICH associates with centromeric DNA and then, in anaphase specifically, with histone‐free threads of DNA called ultrafine bridges (UFBs) that link the separating sister chromatids.^[^ ^20^, ^21^ ^]^ UFBs arise from specific loci, including centromeres,^[^ ^22^ ^]^ common fragile sites (CFSs),^[^ ^23^ ^]^ the ribosomal DNA,^[^ ^19^ ^]^ and telomeres.^[^ ^24^ ^]^ Currently, the only method to reveal UFBs is via detection of specific proteins that bind to them. In addition to PICH, these include BLM, RPA, and RIF1.^[^ ^25^, ^26^, ^27^ ^]^ PICH deficiency in cell lines leads to a modest impairment in chromosome condensation and a defect in the resolution of UFBs.^[^ ^19^ ^]^ In the case of UFBs that arise from CFSs, the FANCD2 and FANCI proteins form foci at the termini of each UFB.^[^ ^28^ ^]^ Occasionally, a faintly stained FANCD2/I “thread” can be seen to connect the strongly stained pair of foci.^[^ ^23^ ^]^ Among the UFB binding proteins, PICH is particularly important in that it is required for the recruitment of the other proteins to UFBs.^[^ ^27^, ^29^ ^]^ A failure to resolve UFBs in a timely manner usually leads to the appearance of markers of DNA damage in the daughter cells,^[^ ^19^ ^]^ which underlines the importance of PICH in genomic stability maintenance. However, the precise function of PICH is still poorly understood, especially its in vivo role under physiological conditions. Previously, Albers et al. constructed a *Pich* conditional knock‐out (cKO) mouse line and revealed that loss of *Pich* induced chromosomal instability and embryonic lethality.^[^ ^30^ ^]^ However, the in vivo function of PICH still remain largely unknown.

In this study, we reveal a key role for PICH in embryonic hematopoiesis in mice. Our data indicate that loss of PICH promotes the expression of cytoplasmic dsDNAs and causes production of type I IFNs mediated by the activation of the cGAS‐STING pathway. Consequently, HSCs from PICH‐defective mice exhibit an impaired reconstitution potential in vitro and in vivo. These data reveal that PICH is essential for embryonic hematopoiesis and provide experimental evidence for a mechanistic link between genomic instability and defective hematopoiesis.

## 2 Results

### 2.1 PICH Is Essential for Embryonic Hematopoiesis in Mice

**Figure 1:**
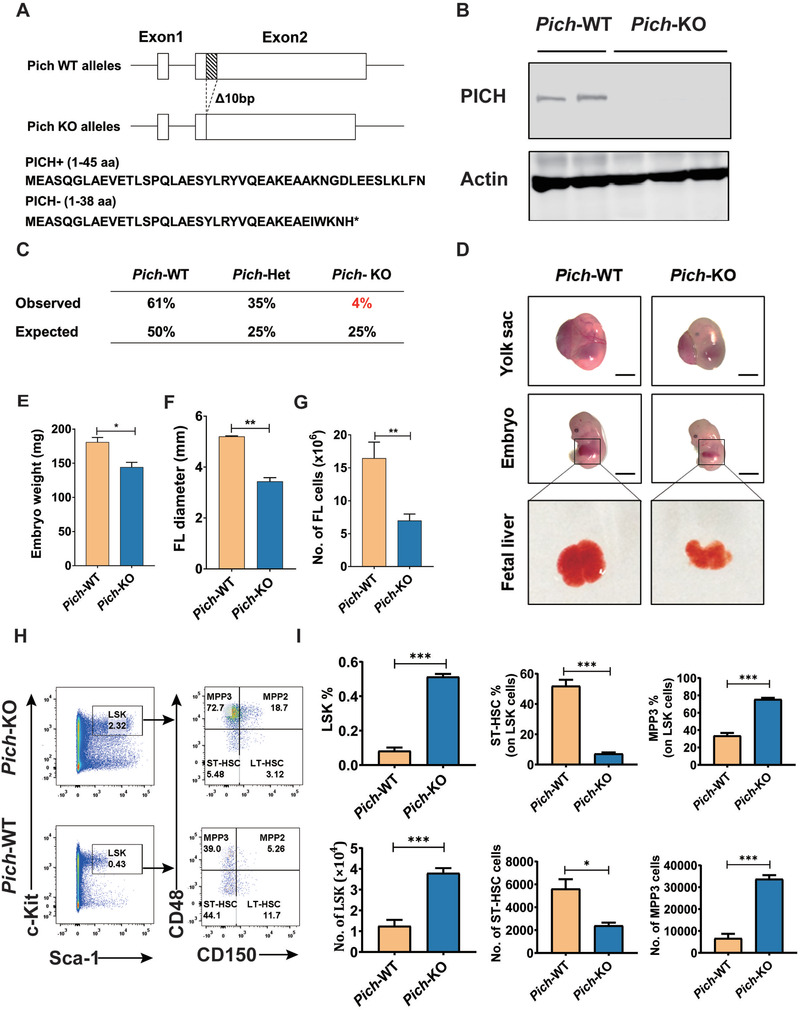
PICH is essential for embryonic hematopoiesis and development in mice. A) Schematic representation of the gene targeting strategy. *Pich* KO mice were generated using TALEN technology wherein 10 bp was deleted from the beginning of Exon 2. This would result in the expression of only a 38 aa truncated PICH peptide. B) Confirmation of the lack of PICH protein expression in the *Pich*‐KO cells, as determined by western blotting analysis of whole tissue protein extracts isolated from WT and *Pich* KO male embryos. Actin was used as a loading control. C) Genotypes of newborn mice from crosses between *Pich* ^+/−^ females and WT male mice, n = 23. D) E14.5 embryos were isolated and visualized under a dissecting microscope. Scale bar = 1 mm. A blow‐up of the fetal liver in each case is shown. E) Quantification of embryo weight. *N* = 3 for each. ∗*p* ≤ 0.05. F) Quantification of fetal liver diameter, n = 3 for each. ∗∗*p* ≤ 0.01. G) Quantification of total cell number in fetal livers, n = 5 for each. ∗∗*p* ≤ 0.01. H) Flow cytometry analysis of FL LSK cells and HSCs in *Pich*‐WT and *Pich*‐KO mice. Lin^−^ cells were gated out for LSK cells analysis. I) Percentages and cell numbers of the indicated cells in FL of WT and *Pich*‐KO mice, n = 4 for each. ∗*p* ≤ 0.05; ∗∗*p* ≤ 0.01; ∗∗∗*p* ≤ 0.001.

To define the in vivo functions of PICH, we constructed a *Pich*‐KO mouse line by deleting ten bases from exon 2 of *Pich*, which is predicted to result in the expression of severely truncated PICH polypeptide lacking all known functional domains (**Figure** **1**A). The KO efficiency of PICH in the cells from KO embryos was confirmed using western blotting (Figure 1B). As the *Pich* gene is located on the X chromosome, there is a single *Pich* allele in male mice, but two alleles in female mice. To generate *Pich*‐KO mice, we crossed *Pich* ^+/−^ female mice with WT male mice. Similar results were found in our mice model to those reported by Albers et al.; however, in our strain a small number of viable *Pich*‐KO mice were born, although the frequency was well below what would be expected according to normal Mendelian inheritance (around 4% of male offspring rather than the expected 25%) (Figure 1C).

Moreover, our data suggested that PICH is required for hematopoiesis. There was a clear difference in appearance and weight of the WT and *Pich*‐KO embryos (Figure 1D,E), as well as in the size and cell content of the FL (Figure 1F,G and Figure S1A, Supporting Information).

To determine the role of *Pich* in embryonic hematopoiesis, FL cells were obtained from *Pich*‐WT or *Pich*‐KO embryos and stained with a combination of slam (CD150, CD48) markers in addition to LSK markers (lineage, Sca‐1, c‐Kit). Even though the total FL cellularity was reduced, the deletion of *Pich* led to a significantly increased absolute number of LSK cells and MPP3 cells (CD150− CD48+ LSK) but a decreased number of ST‐HSCs (CD150− CD48− LSK). No obvious difference was found in the quantity of LT‐HSC (CD150+ CD48− LSK) (Figure 1H,I and Figure S1B, Supporting Information). Moreover, the progenitors’ compartments were also affected by *Pich* deletion. Increased CLP populations and decreased CMP populations were detected in *Pich*‐KO FLs (Figure S1C, Supporting Information). The cell death level of CMP, GMP, and CLP were also elevated in *Pich*‐KO FLs (Figure S1D, Supporting Information). These data suggested that PICH deficiency affected the stem and progenitor cell compartments.

### 2.2 PICH Is Indispensable for the Maintenance of Fetal Liver Hematopoietic Stem Cell Function in Mice

**Figure 2:**
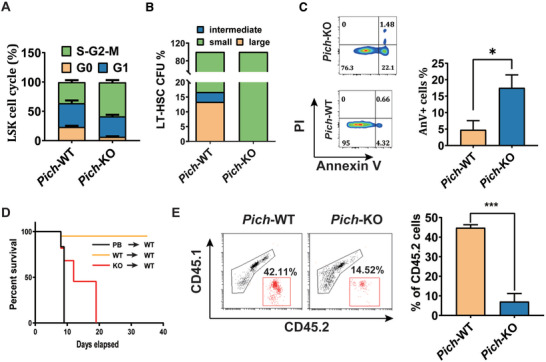
PICH is required for the maintenance of HSC function. A) Cell cycle analysis of the indicated LT‐HSCs through staining with DAPI and Ki67, n = 4 for each. ∗∗∗*p* ≤ 0.001. B) Percentage of large, intermediate, and small colonies from a single colony forming assay of sorted LT‐HSCs. C) Cell death analysis of LSK cells in FL of WT and *Pich*‐KO mice using flow cytometry, n = 3 for each. ∗*p* ≤ 0.05; ∗∗*p* ≤ 0.01; ∗∗∗*p* ≤ 0.001. D) The survival rate of lethally irradiated WT recipients with adopted WT or *Pich* KO fetal liver cells, or PBS as a control. E) Representative FACS plots and percentage of donor‐derived bone marrow cells at 12 weeks after competitive fetal liver cell transplantation, n  for each. ∗∗∗*p* ≤ 0.001.

Through staining for the proliferation marker Ki‐67, we observed that more *Pich*‐KO LT‐HSCs were actively proliferating (**Figure** **2**A). Next, we analyzed the ability of the LT‐HSCs cells to reconstitute a functional hematopoietic system. First, we performed a single‐cell colony formation assay using sorted LT‐HSCs from FLs. After 14‐days in culture, there were no large or intermediate‐sized colonies in *Pich*‐KO group, in contrast to the WT cells (Figure 2B). Moreover, the *Pich*‐KO LSKs showed an increased level of apoptosis compared to WT cells, suggesting the functional insufficiency of *Pich*‐KO HSCs (Figure 2C). To investigate the long‐term effects of *Pich* deletion on FL HSCs in vivo, we transplanted total FL cells into lethally irradiated WT recipients (3 × 10^6^ cells per recipient). However, none of the recipient mice that adopted *Pich* KO liver cells was able to survive beyond 20 days after transplantation (compared to over 35 days for the WT liver cells) (Figure 2D). We then conducted a competitive transplantation analysis by transplanting 5 × 10^4^ whole FL cells from *Pich* KOs or their WT control littermates as donor cells (CD45.2), together with 1 × 10^5^ recipient bone marrow (BM) cells (CD45.1 hereafter), into lethally irradiated recipient mice (Figure 2E). These results indicated that *Pich* KO cells exhibit a significant defect in reconstitution capacity compared to the WT FL cells.

### 2.3 PICH Deficiency Promotes Type I Interferons Production in Fetal Liver Hematopoietic Stem Cells

**Figure 3:**
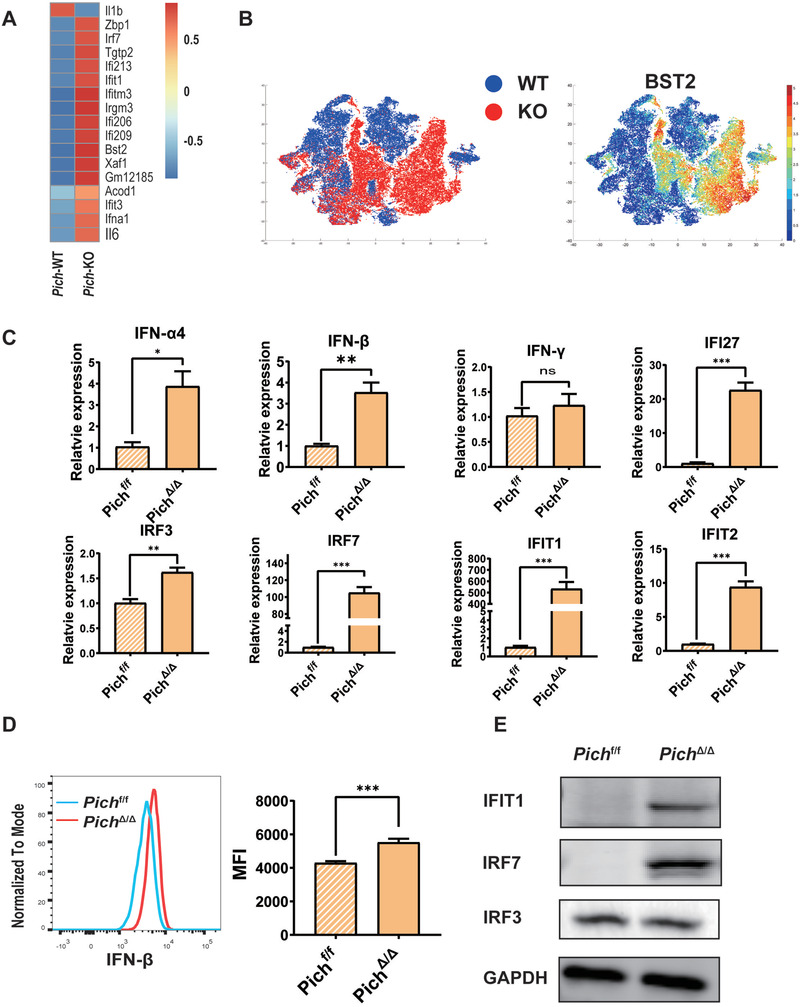
PICH deficiency induces type‐I IFN production. A) WT and *Pich*‐KO LSK cells were subjected to gene expression profiling through RNA sequencing. The enriched IFN‐*β* response gene set is shown. B) WT and *Pich*‐KO FL cells were subjected to mass spectrometry analysis, showing expression of BST2. C) mRNA expression of type‐I IFN genes in *Pich* ^f/f^ (*Pich* ^flox/flox^) and *Pich* ^Δ/Δ^ (conditional knock out of *Pich* in hematopoietic cells using vav‐cre) fetal livers, as analyzed by RT‐PCR, n = 3 for each, n = 3 for each. ∗*p* ≤ 0.05; ∗∗*p* ≤ 0.01; ∗∗∗*p* ≤ 0.001. D) IFN‐*β* levels were determined by FACS in LSKs of *Pich* ^f/f^ and *Pich* ^Δ/Δ^ mice, n   = 7 for each. ∗∗∗*p* ≤ 0.001. E) Protein levels of IFIT1, IRF3, and IRF7 were determined by western blotting in *Pich* ^f/f^ and *Pic*h^Δ/Δ^ FLs.

To determine the mechanism by which PICH deficiency regulates HSCs, we conducted a transcriptome‐wide gene expression analysis of *Pich*‐KO and *Pich*‐WT LSK cells. These RNA sequencing results indicated that IFN‐*β* response genes were more highly expressed in *Pich*‐KO LSK cells than in WT LSK cells (**Figure** **3**A). Additionally, the level of the type I IFN‐sensitive response protein, BST2, was also increased in the *Pich*‐KO FL cells (Figure 3A,B). Given that type I IFNs activate quiescent HSCs and drive their exhaustion,^[^ ^32^, ^33^, ^34^, ^35^ ^]^ we thus focused on how *Pich* deficiency might lead to increased production of type I IFNs. Type I IFN mRNAs were elevated in *Pich* ^Δ/Δ^ FL cells (conditional KO of *Pich* in hematopoietic cells using Vav‐Cre) compared to those of *Pich* ^f/f^ FL cells (Figure 3C), and LSK cells exhibited elevated IFN‐*β* expression after *Pich* deletion (Figure 3D). Moreover, IFN‐*β* content was significantly elevated in LSK cells rather than lineage positive cells or LK cells in *Pich* ^Δ/Δ^ FLs (Figure S2A, Supporting Information). The protein contents of IFIT1 and IRF7 were also elevated after *Pich* deletion (Figure 3E). We also detected increased IRF7expression both in cytoplasm and nuclear in Pich^Δ/Δ^ LSK cells (Figure S2B, Supporting Information). Therefore, we hypothesized that IRF7 mediated the downstream type I IFN signaling in our model.

### 2.4 *Ifnar1* Knock Out Reverses the Hematopoietic Stem Cell Exhaustion Induced by PICH Deficiency

**Figure 4:**
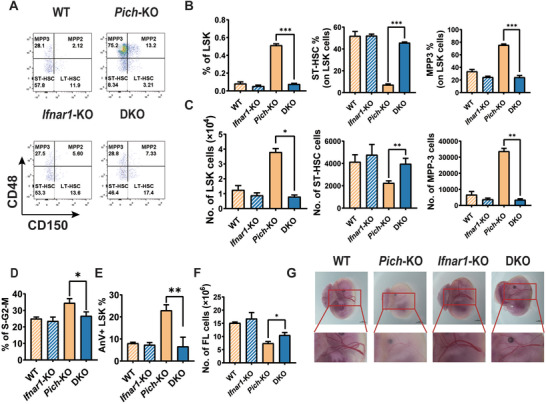
IFNAR1 deletion alleviates the HSC dysfunction in *Pich*‐KO mice. A) Flow cytometry analysis of FL HSCs in WT, *Pich*‐KO, *Ifnar1*‐KO, and DKO mice. Sca‐1^+^c‐Kit^+^ cells in the Lin^−^ cells were gated out for LT‐HSC analysis. B) Percentages of the indicated cells in WT, *Pich*‐KO, *Ifnar1*‐KO, and DKO mice, n = 3 for each. ∗p ≤ 0.05; ∗∗*p* ≤ 0.01; ∗∗∗*p* ≤ 0.001. C) Cell numbers of the indicated cells in WT, *Pich*‐KO, *Ifnar1*‐KO, and DKO mice. D) Cell cycle analysis of LT‐HSCs through staining with DAPI and Ki67, n = 3 for each. ∗*p* ≤ 0.05. E) Cell death analysis of LSK cells in FL of WT and *Pich*‐KO mice using flow cytometry, n = 3 for each. ∗∗*p* ≤ 0.01. F) Quantification of total cell number in FL of WT, *Pich*‐KO, *Ifnar1*‐KO, and DKO mice, n = 3 for each. ∗*p* ≤ 0.05. G) E14.5 embryos were isolated and visualized under a dissecting microscope. Scale bar = 1mm. A blow‐up of the fetal liver in each case is shown.

Type I IFNs exert their proproliferation effect on dormant HSCs via the IFN‐*α*/*β* receptor (IFNAR).^[^ ^32^, ^33^, ^34^, ^35^ ^]^ To further validate the proproliferation action of PICH deficiency‐induced IFN production in HSCs, we generated *Ifnar1* and *Pich* double knock‐out (DKO) embryos via crossing *Pich*‐het (heterozygous) mice with *Ifnar1*‐KO mice (Figure S3A,B, Supporting Information). We noticed that DKO embryos displayed similar numbers of both LSKs and ST‐HSCs compared to WT embryos, whereas *Pich*‐KO embryos still showed a higher number of LSKs and a reduced number of ST‐HSCs compared to WT embryos (**Figure** **4**A–C). The actively cycling state of LT‐HSCs in *Pich*‐KO embryos was reversed by additional KO of *Ifnar1* (Figure 4D). Consequently, the viability of DKO LSK cells was maintained, while ≈25% of *Pich*‐KO LSK cells underwent cell death (Figure 4E). The embryonic anemia in *Pich*‐KO mice was also partially rescued by *Ifnar1* deletion (Figure 4F,G). Taken together, these data indicated that PICH preserved the dormancy of HSCs and embryonic development via suppressing type I IFN signaling.

### 2.5 Increased dsDNA Expression Activates cGAS‐STING Pathway after PICH Deletion

**Figure 5:**
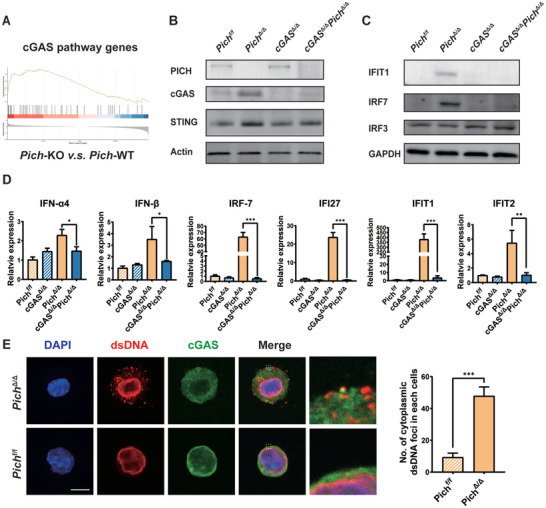
PICH deficiency promotes cGAS‐STING activation. A) WT and *Pich*‐KO LSK cells were subjected to gene expression profiling through RNA sequencing. The enriched cGAS‐STING pathway gene set is shown. B) Protein levels of PICH, cGAS, and STING were determined by western blotting in *Pich* ^f/f^, *Pich* ^Δ/Δ^, *cGAS* ^Δ/Δ^ (conditional knock out of *cGAS* in hematopoietic cells using vav‐cre), and *cGAS* ^Δ/Δ^ *Pich* ^Δ/Δ^ (conditional double knock out of *cGAS and Pich* in hematopoietic cells using vav‐cre) FLs. C) Protein levels of IFIT1, IRF7, and IRF3 were determined by western blotting in *Pich* ^f/f^, *Pich* ^Δ/Δ^, *cGAS* ^Δ/Δ^, and *cGAS* ^Δ/Δ^ *Pich* ^Δ/Δ^ FLs. D) mRNA expressions of type‐I IFN genes in *Pich* ^f/f^, *Pich* ^Δ/Δ^, *cGAS* ^Δ/Δ^, and *cGAS* ^Δ/Δ^ *Pich* ^Δ/Δ^ fetal livers, as analyzed by RT‐PCR, n = 3 for each. ∗*p* ≤ 0.05; ∗∗*p* ≤ 0.01; ∗∗∗*p* ≤ 0.001. E) Immunofluorescence analysis of dsDNAs in LSK cells isolated from E14.5 fetal livers. A representative immunofluorescence image is shown (Scale bar = 10 µm). 100 cells per group. ∗∗∗*p* ≤ 0.001.

cGAS‐STING pathway activation is well defined as a major source of type I IFNs. Of relevance to this, our RNA sequencing data showed that the expressions of cGAS pathway genes were highly elevated in *Pich*‐KO LSK cells, suggesting that cGAS activation might be driving type I IFN production (**Figure** **5**A). Moreover, we observed that cGAS, STING, IFIT1, and IRF7 protein expression were elevated following *Pich* deletion (Figure 5B,C), and that depletion of cGAS suppressed the production of type I interferons (Figure 5B–D and Figure S4A, Supporting Information). PICH is well known to participate in the maintenance of genomic stability, and previous work from Albers et al. showed that loss of PICH induced genomic instability in mouse embryos. Consistently, we found elevated DNA damage level in LSK cells in our model (Figure S4B,C, Supporting Information). Hence, we sought a connection between genomic instability in PICH‐deficient cells and the activation of the cGAS‐STING pathway. Cytoplasmic dsDNA, micronucleus, or chromothripsis are known activators of this pathway. We observe that deletion of *Pich* promoted the accumulation of cytoplasmic dsDNAs that colocalized to a significant degree with cGAS protein (Figure 5E). Taken together, these data suggest that *Pich* deletion promotes the production of type I IFNs via dsDNA‐mediated, cGAS‐STING pathway activation.

### 2.6 cGAS Activation Promotes Hematopoietic Stem Cell Exhaustion in PICH Deficiency Mice

**Figure 6:**
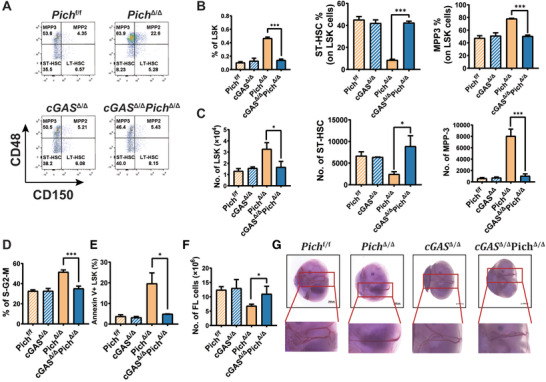
Blocking of cGAS‐STING reversed PICH deficiency‐induced HSC dysfunction. A) Flow cytometry analysis of FL HSCs in *Pich* ^f/f^, *Pich* ^Δ/Δ^, *cGAS* ^Δ/Δ^, and *cGAS* ^Δ/Δ^ *Pich* ^Δ/Δ^ mice. Sca‐1^+^c‐Kit^+^ cells in the Lin^−^ cells were gated out for LT‐HSC analysis. B) Percentages of the indicated cells in *Pich* ^f/f^, *Pich* ^Δ/Δ^, *cGAS* ^Δ/Δ^, and *cGAS* ^Δ/Δ^ *Pich* ^Δ/Δ^ mice, n = 3 for each.∗∗∗*p* ≤ 0.001. C) Cell numbers of the indicated cells in *Pich* ^f/f^, *Pich* ^Δ/Δ^, *cGAS* ^Δ/Δ^, and *cGAS* ^Δ/Δ^ *Pich* ^Δ/Δ^ mice, n = 3 for each. ∗*p* ≤ 0.05; ∗∗∗*p* ≤ 0.001. D) Cell cycle analysis of the indicated LT‐HSCs through staining with DAPI and Ki67, n = 3 for each. ∗*p* ≤ 0.05. E) Cell death analysis of LSK cells in FL of *Pich* ^f/f^, *Pich* ^Δ/Δ^, *cGAS* ^Δ/Δ^, and *cGAS* ^Δ/Δ^ *Pich* ^Δ/Δ^ mice using flow cytometry, n = 3 for each. ∗∗∗*p* ≤ 0.001. F) Quantification of total cell number in FL of *Pich* ^f/f^, *Pich* ^Δ/Δ^, *cGAS* ^Δ/Δ^, and *cGAS* ^Δ/Δ^ *Pich* ^Δ/Δ^ mice, n = 3 for each. ∗*p* ≤ 0.05. G) E14.5 embryos were isolated and visualized under a dissecting microscope. Scale bar = 1 mm. A blow‐up of the fetal liver in each case is shown.

To examine whether PICH deficiency‐induced activation of cGAS occurs in a physiological setting to promote proliferation HSCs, we generated *Pich* ^Δ/Δ^ *cGAS* ^Δ/Δ^ mice by crossing *Pich* ^Δ/Δ^ mice (conditional KO of *Pich* in hematopoietic cells using *Vav*‐Cre) with *cGAS* ^Δ/Δ^ mice (conditional KO of *cGAS* in hematopoietic cells using *Vav*‐Cre). Consistent with our hypothesis, *Pich* ^Δ/Δ^ *cGAS* ^Δ/Δ^ mice showed similar numbers of both LSKs and ST‐HSCs compared to *Pich* ^f/f^ mice, whereas *Pich* ^Δ/Δ^ mice showed a higher number of LSKs and a reduced number of ST‐HSCs in contrast to *Pich* ^f/f^ mice (**Figure** **6**A–C). The proproliferation and the elevated apoptosis characteristic of PICH deficiency were also attenuated by *cGAS* deletion (Figure 6D,E). Finally, the impaired progenitor cell compartments and embryonic anemia seen in *Pich* ^Δ/Δ^ mice were rescued by the additional deletion of *cGAS* (Figure 6F,G and Figure S5A,B, Supporting Information). We conclude, therefore, that the production of type I IFNs mediated by the cGAS‐STING pathway underlies *Pich* deficiency‐induced HSC dysfunction and subsequent embryonic lethality.

## 3 Discussion

In this study, we investigated the role of *Pich* in embryonic hematopoiesis. We observed that a defect in embryonic hematopoiesis associated with *Pich* deficiency is responsible for the embryonic lethality of *Pich* KO mice. We then investigated the mechanism underlying this effect. We determined that elevated type I IFNs activated the cycling of HSCs, reduced the absolute number of ST‐HSCs, promoted the cell death of HSCs, and subsequently impaired the reconstitute ability of HSCs in vitro and in vivo. Deletion of the IFN‐*α*/*β* receptor IFNAR1 reversed the HSCs dysfunction in *Pich*‐KO mice. In addition, we observed that the cGAS‐STING pathway was strongly activated in *Pich*‐KO HSCs, consistent with the finding that the level of cytoplasmic dsDNA (and the colocalization between this cytoplasmic dsDNAs and cGAS) was elevated in the PICH‐deficient HSCs. These findings strongly suggest that the cGAS‐STING pathway is the key upstream signaling pathway that is driving type I IFN production. Indeed, we showed that expression of type I IFNs was reduced after cGAS deletion. Finally, the defective hematopoiesis associated with PICH‐deficient mice was also suppressed after cGAS deletion. Taken together, these data provide the first experimental evidence for a mechanistic link between PICH and hematopoiesis failure.

Our results reveal that PICH is essential for faithful hematopoiesis during embryonic development. However, no difference was found in the cell numbers and functions of bone marrow HSC between survived adult *Pich* ^Δ/Δ^ and *Pich* ^f/f^ mice (Data not show). HSCs in the FL are thought to be highly proliferative; it was estimated that FL HSCs expand by more than 100‐fold within a short period (around 5 days) during embryonic development.^[^ ^4^, ^36^, ^37^ ^]^ By contrast, the adult bone marrow HSCs are generally in a quiescent state.^[^ ^38^, ^39^, ^40^, ^41^, ^42^ ^]^ Replication stress induced in stem cells by repeated rapid cell cycling might be a major source of genome instability in *Pich* KO FL HSCs. Increasing *Rrm2* gene dosage is a well‐defined method to suppress replication stress.^[^ ^43^ ^]^ Thus, we conducted another research by crossing *Pich* het mice with RRM2 transgenic mice. However, the additional two copies of *Rrm2* gene failed to reverse the HSC dysfunctions in *Pich*‐KO FLs (Figure S6A,B, Supporting Information). These data suggested us that two additional copies of Rrm2 gene might not be sufficient to reverse the HSC dysfunction in *Pich*‐KO FLs, or that the cytoplasmic dsDNAs generated in *Pich*‐KO HSCs might not be replication‐dependent. Further research will be required to reveal the source of cytoplasmic dsDNAs in *Pich*‐KO FL HSCs.

Previous in vitro studies using cell line models have shown that PICH plays a key role in the processing of UFBs in anaphase, alongside BLM, TOP3*α*, RMI1, and RMI2. In the absence of PICH, none of these other proteins is able to recognize UFB structures.^[^ ^44^, ^45^, ^46^ ^]^ Deficiency in any of these proteins in cell lines leads to an accumulation of chromosomal abnormalities, including anaphase bridges and micronuclei.^[^ ^45^, ^46^ ^]^ In our study, we detected obvious cytoplasmic dsDNA in Pich‐KO HSCs. Cytoplasmic dsDNA was known to be generated from damaged mitochondria or genomic DNA.^[^ ^47^ ^]^ Considering the functions of PICH in the processing of UFBs, we assumed that the increased cytoplasmic dsDNA in PICH‐KO embryos might be derived from damaged genomic DNA. Interestingly, KO of TOP3*α*, RMI1, BLM, or PICH leads to embryonic lethality in mice.^[^ ^48^, ^49^, ^50^ ^]^ We propose that the lethality due to PICH deficiency induces cGAS‐STING pathway‐mediated type I IFNs production in the FL HSCs, which ultimately is the cause of defective hematopoiesis. Our data extend the connections between mitotic abnormalities and genomic instability in an in vivo setting and reveal that PICH is important for the maintenance of a functional pool of FL HSCs.

AA is a rare and potentially life‐threatening failure of haemopoiesis that results in pancytopenia and hypocellular bone marrow.^[^ ^51^ ^]^ Most cases are acquired sporadically, but there are also some unusual inherited forms. Genome maintenance genes, like FA genes, *DKC1* and *hTERC*, are known to cause BMF.^[^ ^10^, ^12^ ^]^ But those patients rarely respond to immunosuppressive therapies and can only be managed by supportive care or bone‐marrow transplantation in severe cases.^[^ ^52^ ^]^ Better understanding the pathophysiology of HSC failure in FA genes, *DKC1* or *hTERC* deficiency cases is still needed to identify effective therapeutic targets. Two FA genes, FANCD2/I, are consistently associated with PICH‐positive UFBs, suggesting a possible functional interaction between PICH and FANCD2/I.^[^ ^53^ ^]^ We would suggest a further investigation of the role of dsDNA‐cGAS‐type I IFN axis in the pathogenesis of FA is warranted.

Our findings have wider significance for our understanding of how genomic instability impacts on HSC function during embryonic development. Because PICH deficiency induces cGAS‐STING pathway‐mediated type I IFN production, as well as dysregulated embryonic hematopoiesis, our findings might explain the clinical relevance of *Pich* mutations in some cases of acquired AA. Indeed, the same underlying mechanisms might also impact the embryonic lethality induced by deletion of other genes involved in UFB resolution, such as *Blm*, *Rmi1*, or *Top3α*. Thus, we have extended the connections between genomic instability and congenital blood disorders.

## 4 Experimental Section

### Generation of PICH Knock‐Out Mice

Mice used in this work were housed at the laboratory animal center of Zhejiang University, China. All the mouse‐work was conducted in agreement with the experimental Animal Welfare and Ethics Committee of Zhejiang University. *Pich*‐KO mice were constructed by Cyagen Biosciences Inc. (Guangzhou, China). cGAS^flox/flox^ mice were purchased from Nanjing Biomedical Research Institute of Nanjing University (Jiangsu, China). *Pich* ^flox/flox^ mice and RRM2‐tg (expression additional two copies of RRM2) mice were provided by Eliene Albers and Andres Lopez‐Contreras (University of Copenhagen, Denmark). *Ifnar1* KO mice were provided by Prof. Xiaojian Wang (Zhejiang University School of Medicine). Toe clips of newborn mice were collected for DNA extraction, and the genotype was determined using the polymerase chain reaction (PCR).

### Mouse Genotyping

PICH‐KO mice were genotyped using three PCR primers: PICH_F1: (5′‐GCCAAAGAAGCAGCTAAGAATG‐3′); PICH_F2: (5′‐GAAGCCAAAGAAGCAGAGATC‐3′); PICH_R: (5′‐CCAGTCCCATATCATCTGC‐3′). A 300 bp WT fragment was amplified using primer PICH_F1 and PICH_R. A 300 bp KO fragment was amplified using primer PICH_F2 and PICH_R.

Embryo gender was identified by Sry gene PCR using the following primers: SRY_F: 5′‐GCATTTATGGTGTGGTCC‐3′; SRY_R: 5′‐CCAGTCTTGCCTGTATGTGA‐3′.

*Pich* ^f/f^ mice and *Ifnar1‐*KO mice were genotyped as described previously.^[^ ^30^, ^31^ ^]^

*cGAS* ^f/f^ mice were genotyped using two primers: cGAS_F: (5′‐CCAGAATTAGGAAATTAACCCC‐3′); cGAS_R: (5′‐GCCAGGTGACACAACATCC). A 270 bp WT fragment and a 400 bp loxp fragment was amplified using primer.

Vav‐Cre were genotyped using 2primers: vav‐cre_F: (5′‐AGATGCCAGGACATCAGGAACCTG‐3′); vav‐cre_R: (5′‐ATCAGCCACACCAGACACAGAGATC‐3′). A 250 bp Cre fragment was amplified using these two primers.

### Flow Cytometry and Cell Sorting

FLs were dissected from E14.5 embryos and disaggregated into a single cell suspension by pipetting. Cell lineages were stained with purified antibodies purchased from BD Biosciences against the following antigens: CD4, CD8, Gr‐1, B220, and Ter119. Conjugated antibodies (Streptavidin‐APC‐Cy7, c‐Kit‐APC, Sca‐1‐PE‐Cy7, CD150‐PerCP‐Cy5.5, and CD48‐FITC) were purchased from Biolegend. DAPI was used to distinguish live cells from dead cells. Cell sorting was performed using a BD Influx cell sorter.

### Immunofluorescence Staining

LSK cells from E14.5 FLs were sorted using BD Influx cell sorter and were immobilized on coverslips using cytospin. Cells were fixed in 4% paraformaldehyde for 15 min at room temperature (RT), and permeabilized with 0.5% Triton‐X100 (Sigma‐Aldrich, V900502) for 20 min at RT. After blocking with 5% bovine serum albumin (Sigma‐Aldrich, #B2064) for 40 min at RT, the cells were incubated with the corresponding primary antibodies for 10 h at 4 °C. They were then incubated with Alexa Fluor 555 or Fluor 488‐conjugated secondary antibodies against rabbit or mouse immunoglobulin G heavy and light chain (Invitrogen, A21424) for 1 h at room temperature. The nucleus was counterstained with DAPI for 10 min. Fluorescent images were captured with an automated Nikon Elipse Ni microscope or SIM microscope with Nikons‐Elements software (Nikon instruments). Antibodies against cGAS (Proteintech, 26416), dsDNA (abcam, ab27156), and IRF7 (Cell Signaling Technology, 72073S) were used.

### Western Blot Assay

FLs were dissected from E14.5 embryos and the lysates were prepared with RIPA buffer (Beyotime, P0013B) containing protease (Roche Diagnostics GmbH, 04‐693‐116‐001) and phosphatase inhibitors (Roche Diagnostics GmbH, 04‐906‐837‐001). The supernatants of cell lysates were run on gels and incubated with relevant antibodies using standard methods. Actin and GAPDH was used as a loading control. Primary antibodies used for western blot as follows: Actin (Santa Cruz Biotechnology, sc‐130300), GAPDH (Proteintech, 60004), PICH (Cell Signaling Technology, 8886), cGAS (Cell Signaling Technology, 31659), STING (Cell Signaling Technology, 50907), IFIT1 (Origene, TA500948), IRF3 (Cell Signaling Technology, 4302S), and IRF7 (Cell Signaling Technology, 72073S).

### Mass Cytometry Analysis

Total FL cells were collected and incubated with TruStain fcX (Biolegend) for FC blocking. After being washed twice with staining buffer (PBS containing 1% bovine serum albumin and 0.05% sodium azide), the FL cells were stained with a mixture of metal‐tagged antibodies (see Table S1, Supporting Information, for the complete antibodies list). All antibodies were conjugated using the MAXPAR reagent (Fluidigm Inc.). Rhodium (1:2000; Fluidigm Inc.) was added to the cells for the last 20 min of staining. Cells were fixed with 1.6% PFA (Sigma‐Aldrich) in PBS and stained with iridium (Fluidigm Inc.).The samples were analyzed on a CyTOF III machine (Fluidigm Inc.). Acquired data were processed using a Cytobank web server (Cytobank Inc.). Rhodium (Rh) and iridium (Ir) (Fluidigm Inc.) intercalators were used to identify live/dead cells.

### Colony Formation Assays

LT‐HSCs sorted from FLs were seeded into 96‐well plate (one cell per well) and the cells were cultured for 2 weeks in liquid medium supplemented with 10% FBS, 100 U mL^−1^ penicillin/streptomycin, 2 mm L‐glutamine, 5 × 10^−5^ *β*‐ME, 10 ng mL^−1^ SCF, TPO, and IL‐13. The resulting cell colonies were classified into three classes: large (containing >10 000 cells), intermediate (between 1000 and 10 000 cells), and small (<1000 cells).

### Bone Marrow Transplantation

CD45.1 recipient mice were irradiated with a lethal dose (8 Gy) of X‐rays. For normal transplantation assays, 3 × 10^6^ total FL cells (CD45.2) were injected intravenously via the tail. For competition transplantation analyses, 5 × 10^4^ CD45.2 FL cells and 1 × 10^5^ CD45.1 BM cells were injected into CD45.1 recipient mice.

### Quantitative‐Polymerase Chain Reaction Analysis

Total mRNA was extracted from FL cells following the manufacturer's instructions. RNA was reverse‐transcribed to cDNA with the PrimeScript RT reagent Kit (TAKARA), and Q‐PCR was performed on a StepOnePlue Real‐Time PCR system (Applied Biosystems) with SYBR Premix Ex Taq (TAKARA), all according to the manufacturers' manuals. The mouse primers used for the Q‐PCR were as follows:

Actin:

Forward: 5′‐GTCCACCGTGTATGCCTTCT‐3′,

Reverse: 5′‐CTCCTGGTGTCCGAACTGAT‐3′;

IFN*α*4:

Forward: 5′‐TGATGAGCTACTACTGGTCAGC‐3′,

Reverse: 5′‐GATCTCTTAGCACAAGGATGGC‐3′;

IFN*β*:

Forward: 5′‐CAGCTCCAAGAAAGGACGAAC‐3′,

Reverse: 5′‐GGCAGTGTAACTCTTCTGCAT‐3′;

IFN*γ*:

Forward: 5′‐ATGAACGCTACACACTGCATC‐3′,

Reverse: 5′‐CCATCCTTTTGCCAGTTCCTC‐3′;

IFIT1:

Forward: 5′‐CTGAGATGTCACTTCACATGGAA‐3′,

Reverse: 5′‐GTGCATCCCCAATGGGTTCT‐3′;

IFIT2:

Forward: 5′‐AGTACAACGAGTAAGGAGTCACT‐3′,

Reverse: 5′‐AGGCCAGTATGTTGCACATGG‐3′;

IFI27:

Forward: 5′‐TTCCCCCATTGGAGCCAAG‐3′,

Reverse: 5′‐AGGCTGCAATTCCTGAGGC‐3′;

IRF3:

Forward: 5′‐GAGAGCCGAACGAGGTTCAG‐3′,

Reverse: 5′‐CTTCCAGGTTGACACGTCCG‐3′;

IRF7:

Forward: 5′‐GAGACTGGCTATTGGGGGAG‐3′,

Reverse: 5′‐GACCGAAATGCTTCCAGGG‐3′;

### Statistical Analysis

GraphPad Prism 7.0 software was used for statistical analysis, and results were displayed as the means ± SEM. The unpaired and two‐tailed *t*‐test was employed for the comparisons between two groups, while analysis of one‐way ANOVA with Sidak's multiple comparisons test for comparing multiple experimental groups with a single control group. *p* values of <0.05 were considered to be statistically significant. *p* values were indicated above the graphs: ∗*p* ≤ 0.05; ∗∗*p* ≤ 0.01; ∗∗∗*p* ≤ 0.001.

## Conflict of Interest

The authors declare no conflict of interest.

## Supporting information

Supporting Information

## Data Availability

The data that support the findings of this study are openly available in [GEO] at https://www.ncbi.nlm.nih.gov/geo/query/acc.cgi?acc=GSE188359, reference number 188359.
