# Supplementary material for: PICH Supports Embryonic Hematopoiesis by Suppressing a cGAS‐STING‐Mediated Interferon Response
Source: Adv Sci (Weinh). 2022 Jan 17;9(7):2103837. doi: 10.1002/advs.202103837 (PMC8895048; doi:10.1002/advs.202103837)
Supplement: Supplementary file 1 — Supporting Information [file ADVS-9-2103837-s001.pdf]

ADVANCED SCIENCE
Open Access

Supporting Information

for *Adv. Sci.*, DOI: 10.1002/advs.202103837

PICH supports embryonic hematopoiesis by suppressing a cGAS-STING-mediated interferon response

Xinwei Geng, Chao Zhang, Miao Li, Jiaqi Wang, Fang Ji, Hanrong Feng, Meichun Xing, Fei Li, Lingling Zhang, Wen Li, Zhihua Chen, Ian D. Hickson, Huahao Shen*, and Songmin Ying**

Supplementary Materials

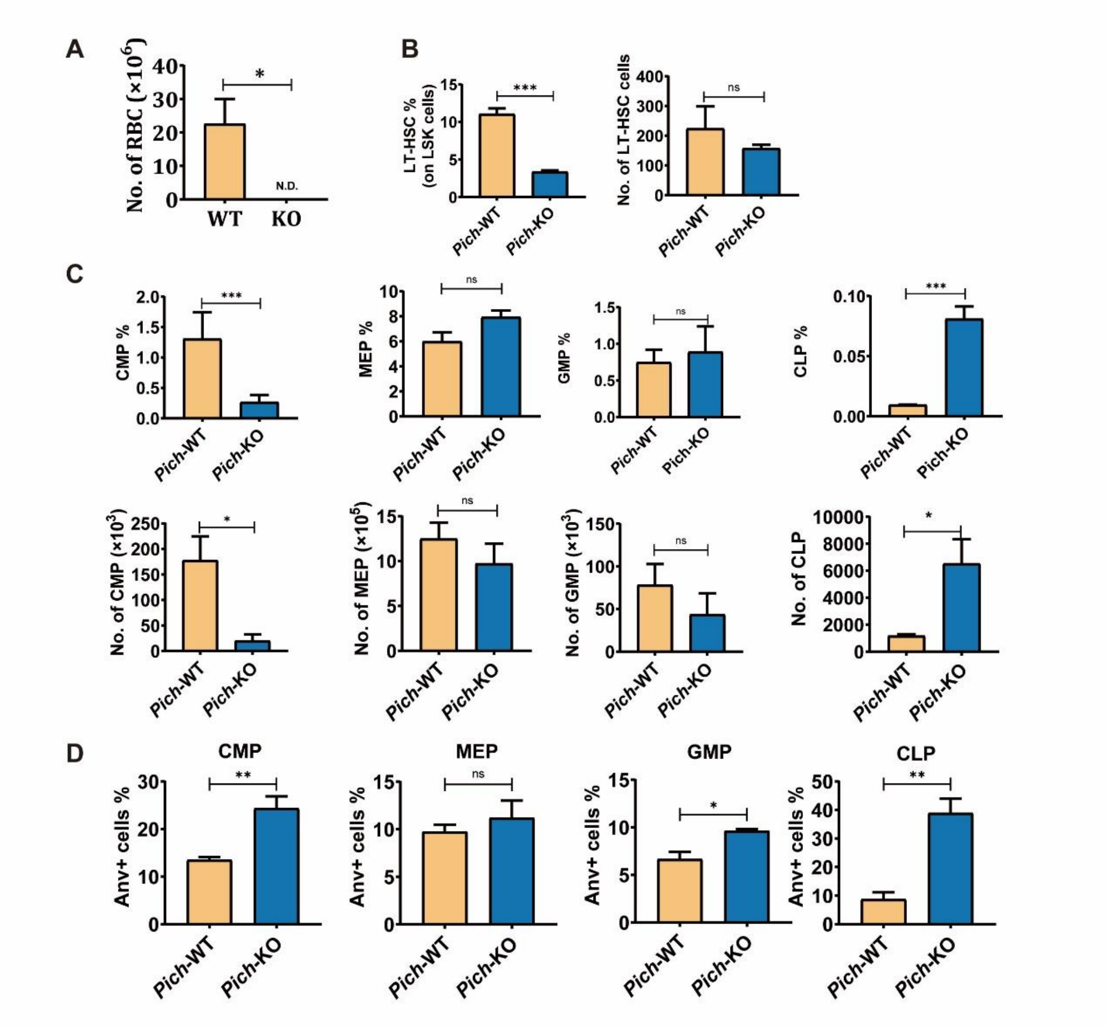

Fig. S1 PICH deficiency affects progenitor cell compartments. (A). The status of red blood cells in the peripheral blood was analyzed via blood routine examination. N=4 for each genotype. $*p \leq 0.05$. N.D. means lower than detection threshold. (B). Percentages and cell numbers of LT-HSCs in *Pich*-WT and *Pich*-KO mice. N=3 for each. $***p \leq 0.001$. (C). Percentages and cell numbers of the indicated cells in *Pich*-WT and *Pich*-KO mice. N=3 for each. $*p \leq 0.05$; $***p \leq 0.001$. (D). Cell death analysis of CMP, MEP, GMP and CLP cells in FL of WT and Pich-KO mice using flow cytometry. N=3 for each. $*p \leq 0.05$; $**p \leq 0.01$.

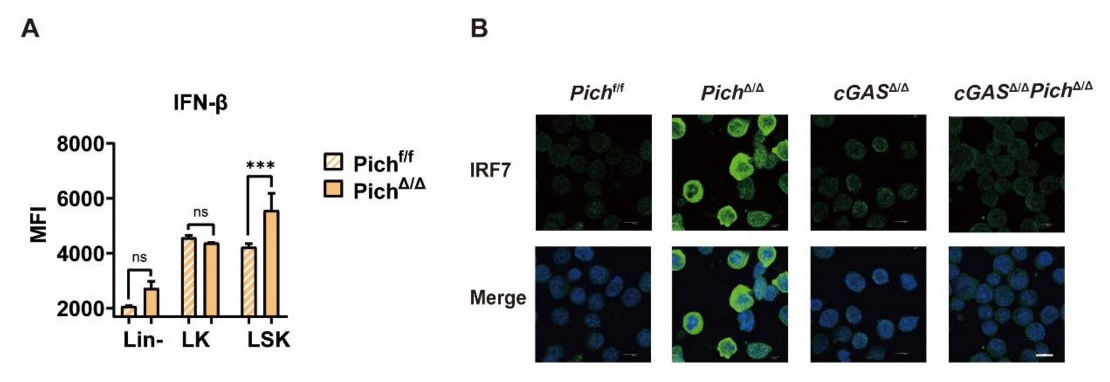

Fig. S2 Type I IFNs activation in *Pich*-KO hematopoietic stem cells. (A). IFN-β levels were determined by FACS in Lin+, LK and LSK cells of WT and *Pich*-KO mice. N=5 for each. $***p \leq 0.001$. (B). Immunofluorescence staining of IRF7 in LSK cells isolated from E14.5 fetal livers. Scale bar=10 μm.

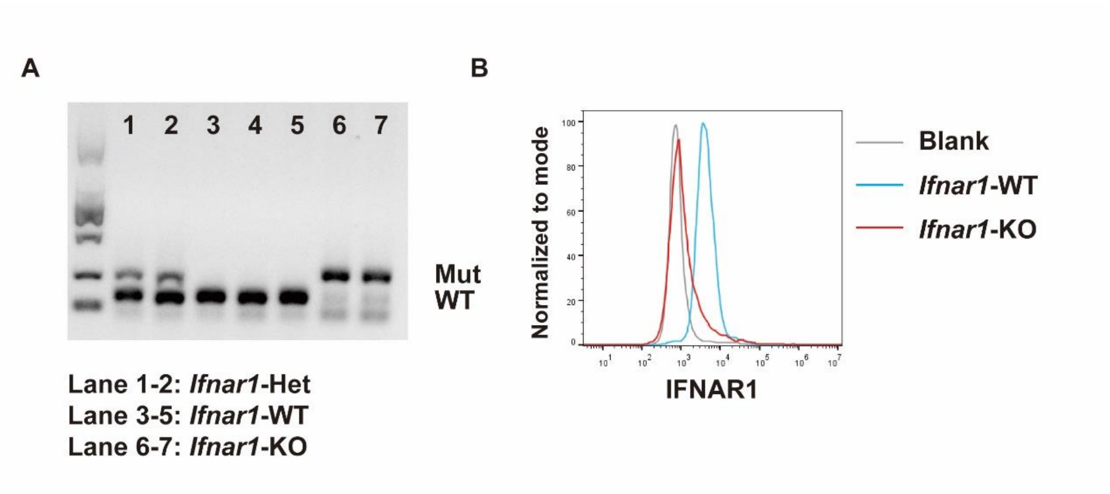

Fig. S3 *Ifnar1* knockout efficiency analysis. (A). Image of *Ifnar1* genotyping PCR products post DNA gel electrophoresis. (B). Surface *Ifnar1* expression levels were determined by FACS in WT and *Ifnar1*-KO spleen cells.

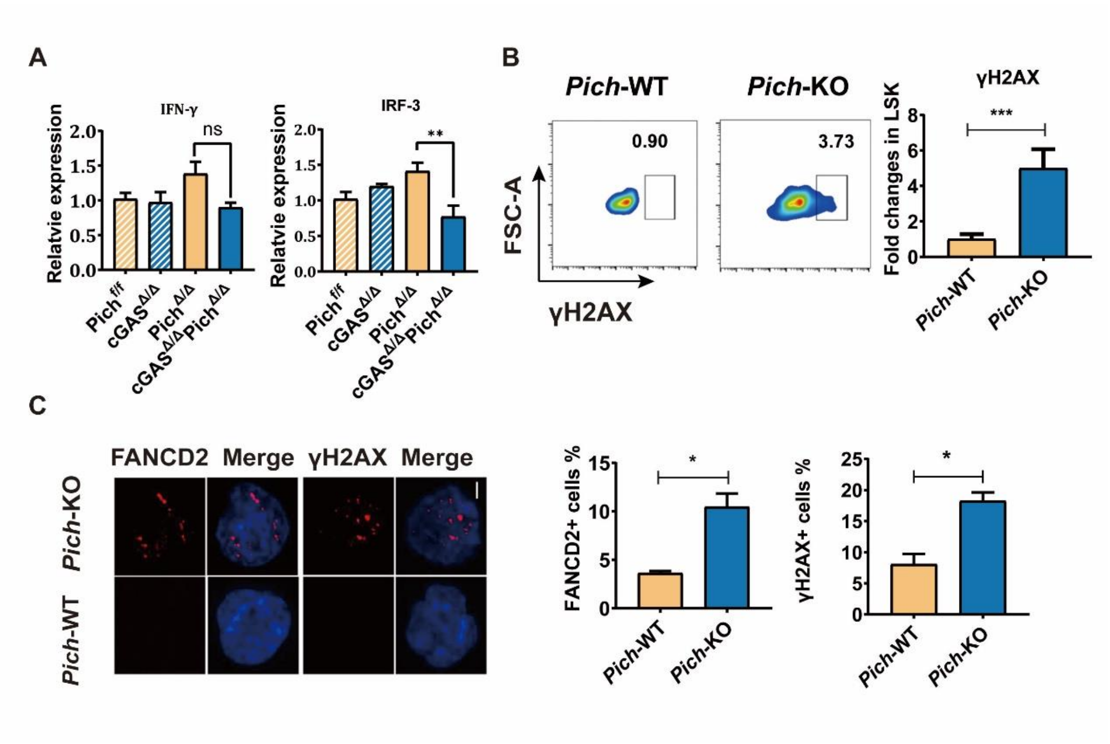

Fig. S4 PICH deficiency induced DNA damage in hematopoietic stem cells. (A). mRNA expression of IFN-γ and IRF-3 in *Pich*$^{f/f}$, *Pich*$^{\Delta/\Delta}$, *cGAS*$^{\Delta/\Delta}$ and *cGAS*$^{\Delta/\Delta}$*Pich*$^{\Delta/\Delta}$ fetal livers, as analyzed by RT-PCR. N=3 for each. N=3 for each. $**p \leq 0.01$. (B). γ-H2AX levels were determined by FACS in LSKs of WT and *Pich*-KO mice. N=4 for each. $***p \leq 0.001$. (C). Immunofluorescence analysis of γ-H2AX and FANCD2 foci in LSK cells isolated from E14.5 fetal livers. Along with quantification of the % cells displaying more than 5 foci for FANCD2 and γ-H2AX. N=3 for each. $*p \leq 0.05$.

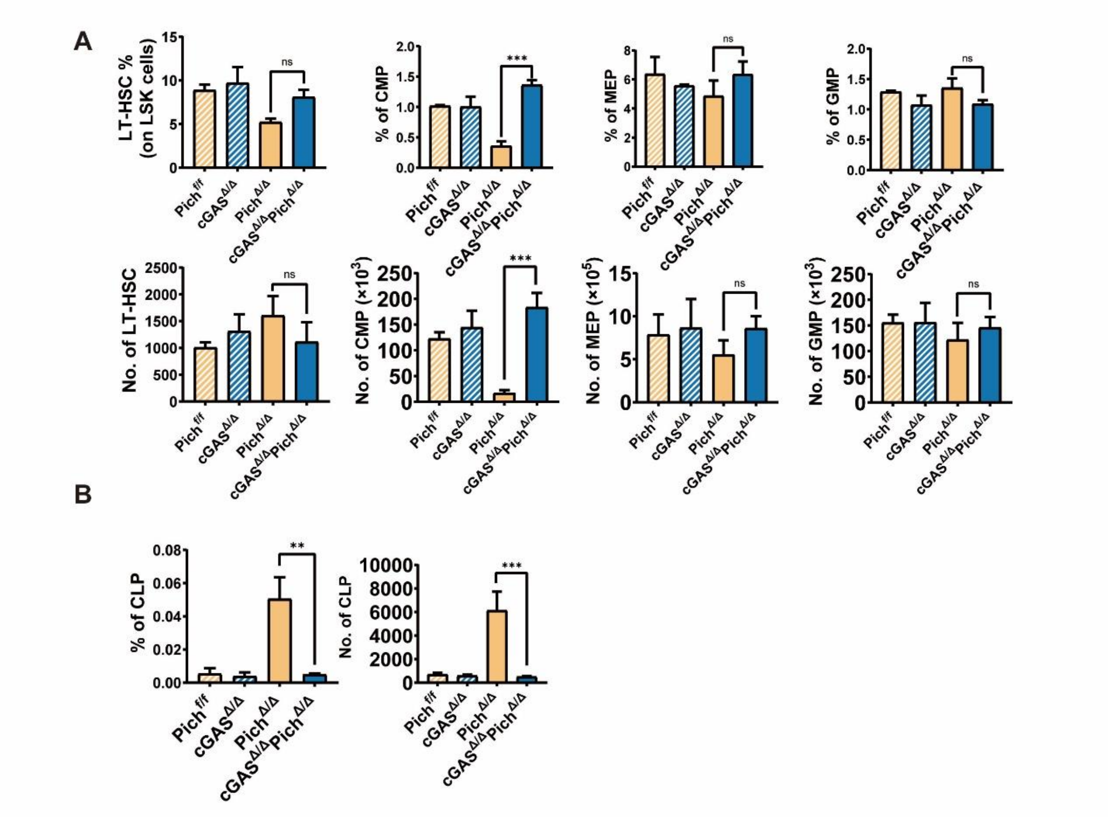

Fig. S5 Deletion of cGAS reversed the decreased CMP and increased CLP populations in PICH deficiency embryos. (A). Percentages and cell numbers of the indicated cells in *Pich*$^{f/f}$, *Pich*$^{\Delta/\Delta}$, *cGAS*$^{\Delta/\Delta}$ and *cGAS*$^{\Delta/\Delta}$*Pich*$^{\Delta/\Delta}$ mice. N=3 for each. $*p \leq 0.05$; $**p \leq 0.01$; $***p \leq 0.001$. (B). Percentages and cell numbers of CLP in *Pich*$^{f/f}$, *Pich*$^{\Delta/\Delta}$, *cGAS*$^{\Delta/\Delta}$ and *cGAS*$^{\Delta/\Delta}$*Pich*$^{\Delta/\Delta}$ mice. N=3 for each. $**p \leq 0.01$; $***p \leq 0.001$.

A

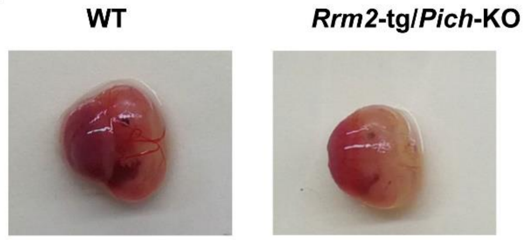

B

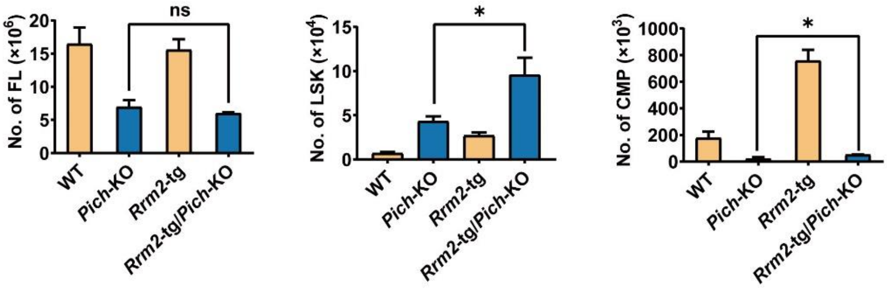

Fig. S6 Reduce replication stress by overexpressing RRM2 fails to alleviate PICH deficiency-induced HSC dysfunction. (A). E14.5 embryos were isolated and visualized under a dissecting microscope. (B). Cell numbers of indicated cells in FL of WT, *Pich*-KO, *Rrm2*-tg and *Rrm2*-tg/*Pich*-KO mice. N=3 for each. $*p \leq 0.05$.

Table S1: Mass cytometry panel

List	mass tag	Marker	Clone No.	Company
1	89Y	CD45	30-F11	Fluidigm
2	115In	CD3e	145.2C11	Biolegend
3	139La	KI67	SolA15	eBioscience
4	142Nd	MHC II	Y3P	Bio-Xcell
5	143Nd	B220	RA3-6B2	Biolegend
6	144Nd	CD16/32	93	Fluidigm
7	146Nd	CD38	90	Biolegend
8	147Sm	Ly6G	IA8	Biolegend
9	148Nd	Ly6C	HK1.4	Biolegend
10	151Eu	Flt3/CD135	113308	R&D
11	152Sm	CD11c	N418	Biolegend
12	153Eu	SiglecF	E50-2440	BD
13	154Sm	CD48	HM48-1	Fluidigm
14	156Gd	BST2	44E9R	RD
15	157Gd	CD25	3C7	Biolegend
16	158Gd	Ter119	Ter119	Biolegend
17	159Tb	F4/80	C1:A3-1	BioRAD
18	161Dy	CD105/Endoglin	209701	R&D

19	164Dy	Ly-6A/E(Sca-1)	D7	Fluidigm
20	165Ho	CD90	T24/31	Bio-Xcell
21	166Er	c-kit/CD117	2B8	Fluidigm
22	167Er	CD150	TC15	Fluidigm
23	169Tm	CD127	A7R34	Biolegend
24	171Yb	CD41	MWReg30	Biolegend
25	172Yb	CD71	R17217	eBioscience
26	173Yb	CD172a	P84	Biolegend
27	174Yb	CD9	KMC8	Biolegend
28	176Yb	IgM	RMM-1	Biolegend
29	197gd	CD4	RM4-5	Biolegend
30	198pt	CD8a	53-6.7	Biolegend
31	209Bi	CD11b	M1/70	homemade
